# Decreasing but differential trends of adverse events among dialysis outpatient following re-enforcement of infection control measures; 20-month surveillance study

**DOI:** 10.1186/1753-6561-5-S6-P208

**Published:** 2011-06-29

**Authors:** HH Balkhy, A El-Saed, F Al Hejaili, M Sallah, A Azzam, A Sayyari

**Affiliations:** 1 Infection Prevention and Control Department, KAMC, Riyadh, Saudi Arabia; 2 Infection Control Department, KAMC, Riyadh, Saudi Arabia; 3 Department of Medicine, KAMC, Riyadh, Saudi Arabia; 4 KAMC, Riyadh, Saudi Arabia; 5 Department of Medicine, KAMC, Riyadh, Saudi Arabia

## Introduction / objectives

We set to monitor adverse events among hemodialysis patientsfollowing re-enforcement of infection control measures.

## Methods

We conducted a 20-month prospective surveillance study among end-stage kidney disease patients served by the outpatient hemodialysis unit at KAMC. We used the same methodology described by US National Healthcare Safety Network (NHSN). We monitored the following adverse events; overnight hospital stay for any reason, outpatient start of an intravenous (IV) antimicrobial, and access-associated bacteremia. Starting the fourth month of the study, we re-enforced infection control measures including aseptic technique, catheter care, hand hygiene, judicious use of antimicrobials, and patient education.

## Results

A total of 339 hospitalizations, 302 antimicrobial start and 174 access-associated bacteremias were observed during 20 months of surveillance. The overall adverse events rate per 100 patient-months showed a 40% decline, p for trend <0.001. For adverse events, the decline was obvious in hospitalizations (39% declined from 10.4 to 6.4, p for trend <0.001) and antimicrobial start (46% declined from 11.7 to 6.4, p for trend <0.001) more than access-associated bacteremia (19% declined from 4.4 to 3.6, p for trend 0.564). Primary (57%) rather than recurrent (2%) adverse events were the main to benefit from the decline. Adverse events associated with central line catheter (38%) declined more than those associated with AV fistula (27%).

## Conclusion

We report a significant decline of overall adverse events in our hemodialysis patients concomitant with enforcement of infection control measures.

## Disclosure of interest

None declared.

